# Histone Deacetylase HDT1 is Involved in Stem Vascular Development in Arabidopsis

**DOI:** 10.3390/ijms20143452

**Published:** 2019-07-13

**Authors:** Yongzhuo Zhang, Bin Yin, Jiaxue Zhang, Ziyi Cheng, Yadi Liu, Bing Wang, Xiaorui Guo, Xiatong Liu, Di Liu, Hui Li, Hai Lu

**Affiliations:** 1 College of Biological Sciences and Biotechnology, Beijing Forestry University, Beijing 100083, China; 2 National Engineering Laboratory for Tree Breeding, Beijing Forestry University, Beijing 100083, China

**Keywords:** HDT1, vascular tissue, stem, secondary cell wall

## Abstract

Histone acetylation and deacetylation play essential roles in eukaryotic gene regulation. HD2 (HD-tuins) proteins were previously identified as plant-specific histone deacetylases. In this study, we investigated the function of the *HDT1* gene in the formation of stem vascular tissue in *Arabidopsis thaliana*. The height and thickness of the inflorescence stems in the *hdt1* mutant was lower than that of wild-type plants. Paraffin sections showed that the cell number increased compared to the wild type, while transmission electron microscopy showed that the size of individual tracheary elements and fiber cells significantly decreased in the *hdt1* mutant. In addition, the cell wall thickness of tracheary elements and fiber cells increased. We also found that the lignin content in the stem of the *hdt1* mutants increased compared to that of the wild type. Transcriptomic data revealed that the expression levels of many biosynthetic genes related to secondary wall components, including cellulose, lignin biosynthesis, and hormone-related genes, were altered, which may lead to the altered phenotype in vascular tissue of the *hdt1* mutant. These results suggested that *HDT1* is involved in development of the vascular tissue of the stem by affecting cell proliferation and differentiation.

## 1. Introduction

The vascular system connects the leaves and other organs with the roots in vascular plants, which is not only essential for supporting the plant’s body structure but also for transporting water, nutrients, and signaling components. The vascular tissues are comprised of xylem and phloem with a layer of cambial cells in between. The vascular cambium is characterized by its multipotent stem cell identity and continued proliferation and differentiation into new xylem and phloem cells during vascular development [1].

Molecular and genetics studies have revealed that hormone and transcription factors regulate vascular tissue proliferation and differentiation. Auxin induces the formation and differentiation of primary cambium cells by directing polar auxin gradients, ultimately enabling plants to develop functional vascular bundles [2]. Exogenous auxin promotes the formation and secondary growth of vascular cambium through polar transport [3]. Auxin signaling interacts with the strigolactone signaling pathway to regulate cambium activity [4]. In addition, many transcription factors are involved in the process of cambium cell differentiation, including HD-ZIP III [5,6], NAC [7,8,9], and MYB [10,11,12,13], which are specifically expressed in vascular tissue to regulate cambium activity and vascular development.

The dynamics of chromatin structure are related to gene expression and play a role in regulating cell division and differentiation [14]. The basic structural unit of chromatin in eukaryotes is the nucleosome containing 147 bp of DNA wrapped around an octamer of four core histones (H3, H4, H2A, H2B). The core histone tail extending from the surface of the nucleosomes provides a site for post-translational modification. Histone modifications, including acetylation, methylation, phosphorylation, ubiquitination, sumoylation, and ADP ribosylation, play a major role in epigenetic regulation of gene expression [15,16]. Histone transferase (HAT) can transfer the acetyl groups on acetyl-CoA to the lysine residues at the end of the histone while HDAC removes the acetyl groups at the end of the histone [17]. Hyper-acetylation of histones is associated with transcriptional activation by loosening the chromatin structure to weaken the interaction between histones and DNA, whereas hypo-acetylation of histones is associated with transcriptional repression by compacting chromatin structure [18].

HDAC is classified into three different families: The RPD3/HDA1 superfamily, the sirtuin family, and the HD2 family [19]. Members of the RPD3/HDA1 superfamily and the sirtuin family are homologous to yeast reduced potassium dependency 3 (RPD3)/HDA1 and silent information regulator 2 (sir2), respectively, whereas the HD2 family proteins are plant-specific HDACs originally identified in maize [20]. In plants, HDACs are involved in gene silencing, stress response, and plant growth processes, such as seed embryogenesis, vegetative growth, morphogenesis, flowering, and aging. In *Arabidopsis*, 18 HDACs and 4 HD-tuins (HDT1–4) have been identified [21]. HD2C has been found to interact with the RPD3-type HDAC, HDA6, and regulates ABA-responsive gene expression in response to ABA and salt stress [22]. AtHDA7 is required for female gametophyte and embryo development in *Arabidopsis* [23]. An *hda9* mutant displayed reduced seed dormancy and faster germination than wild-type plants [24]. Meanwhile, *athda19* mutant plants exhibited dwarfing, leaf malformation, and asymmetric leaf growth [25]. In *Arabidopsis* leaves, HDT1 and HDT2 control leaf polarity by regulating miR165/166 expression through independent pathways of AS1 (ASYMMETRIC LEAVES1) and AS2 [26]. HDT1 and HDT2 fine-tune gibberellin (GA) metabolism and control the switch from cell division to expansion, leading to changes in cell numbers and the root-growth rate [27]. Studies have shown that the transition from cell division to expansion in *Arabidopsis* roots is accompanied by changes in histone acetylation levels [26]. Some studies have shown that *HD2* is expressed in plant stems [28]. In rice, downregulation of *OsHDT702*, a gene homologous with *HDT3*, resulted in changes in acetylation levels, and the leaves and stems of the plant were narrowed [29], which suggests that histone deacetylase plays an important role in regulating stem development. However, it is unclear how histone deacetylases affect vascular tissue development by regulating gene expression.

To investigate the role of HDT1 in vascular development, we characterized the phenotype and gene expression of the *hdt1* mutant. Our results showed that the cell size and number as well as the thickness of the secondary cell wall were altered in the xylem cells. Transcriptomic analysis revealed that many genes involved in cell proliferation and wood formation are differentially expressed in the *hdt1* mutant. This study established the relationship between *HDT1* and vascular tissue proliferation and differentiation in the secondary growth underlying stem development. The role of histone deacetylases in stem growth and development is discussed in this study.

## 2. Results

### 2.1. Characterization of HDT1 Expression and Morphological Analysis of the hdt1 Mutant in Arabidopsis

**Figure 1:**
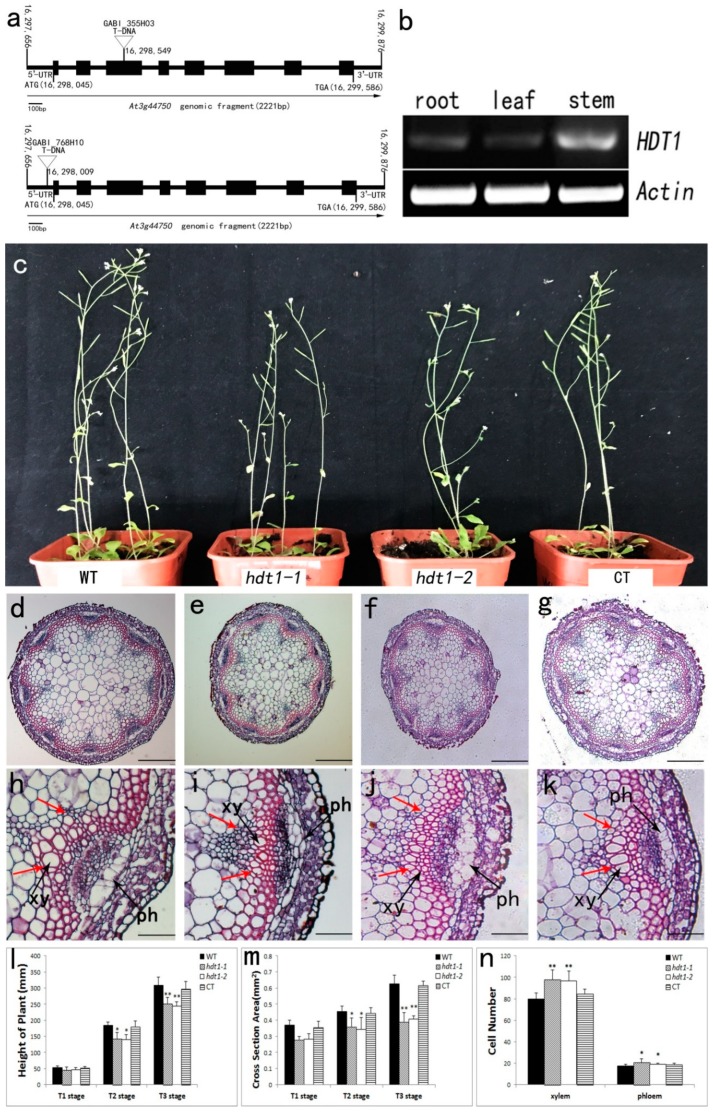
T-DNA insertion mutant and *HDT1* gene expression analyses. Morphological analysis of the *hdt1* mutant and wild-type plants. T1 stage: bolting begins to form the primary stem. T2 stage: the plant has bolted and is flowering. T3 stage: all siliques have formed and the stems have turned yellow. (**a**) Structure of T-DNA insertion sites in *hdt1-1* and *hdt1-2*. Exons are represented by black filled boxes. (**b**) *HDT1* spatial expression analyses by reverse transcription-polymerase chain reaction (RT-PCR). (**c**) Plant growth status in the T2 stage. (**d**–**g**) Cross sections of stems at low magnification in the T3 stage. (**d**), WT; (**e**), *hdt1-1*; (**f**), *hdt1-2*; and (**g**), CT line. Scale bars = 200 μm (**d**–**g**). (**h**–**k**) Cross sections of stems at high magnification view in the T3 stage. (**h**), WT; (**i**), *hdt1-1*; (**j**), *hdt1-2*; and (**k**), CT line. The xylem (xy) and phloem (ph) are indicated. Double red arrows indicate areas that were measured. Scale bars = 50 μm (**h**–**k**). (**l**) Height of stems during different developmental stages. (**m**) Cross-sectional areas of stems during different developmental stages. (**n**) Total number of xylem and phloem cells in a vascular bundle in the T3 stage. (Red double arrows show the area of cells. Data presented are the means of three replicates ± SD. **, *p*-value < 0.01; *, *p*-value < 0.05).

To identify the function of HDT1 during *Arabidopsis* stem development, we obtained two T-DNA insertion mutant lines, named *hdt1-1* (GABI_355H03) and *hdt1-2* (GABI_768H10). T-DNAs are inserted into the 5′ untranslated region and the third exon of *HDT1* in these mutants, respectively (Figure 1a). RT-PCR analysis was performed to characterize the *HDT1* expression pattern by extracting total RNA from various tissues. These data showed that *HDT1* was expressed in all tested tissues, including roots, stems, and leaves, and its expression level was higher in stems than in leaves and roots (Figure 1b). Our data are consistent with previous reports, which showed that *HDT1* is expressed globally in flowers, stems, leaves, young siliques, ovules, embryos, the shoot apical meristem, and primary leaves [30].

### 2.2. Morphological Analysis of the hdt1 Mutants

We did not observe any morphological differences in flowers and leaves between the *hdt1* mutant and wild-type plants growing under the same growth conditions. However, the stems were shorter and thinner in the *hdt1* mutants compared with wild-type plants (Figure 1c–f). By measuring plant height at three different developmental stages from the beginning of bolting (T1 stage) to stem maturation (T3 stage), we found that the height of stems from the T1 stage to T3 stage was shorter in *hdt1* mutant plants than in wild-type plants. At the T3 stage, when plants stop elongating, the *hdt1-1* and *hdt1-2* plants were 18.19% and 20.66% shorter than wild-type plants (Figure 1c,l). The height of the *hdt1* complementation transgenic (CT) plants was not altered compared with that of wild-type plants.

To investigate whether the cell morphology of the stem was affected by *HDT1* mutation, the basal nodes of stems from the T1 to T3 stages were selected for embedding in paraffin sections followed by analysis of the anatomical details of the vascular tissue (Figure 1d–k). By measuring the cross-sectional area of paraffin sections of stems, we found that the stem thickness of *hdt1* mutants decreased significantly compared to wild-type plants at various stages of stem development (T1–T3 stages). The data showed that the stem cross-sectional area of the *hdt1-1* and *hdt1-2* mutant was 34.74% and 37.36% smaller than that of the wild-type at the T3 stage (Figure 1d–g,m). To determine whether this decrease in stem thickness was caused by a decreased cell number or cell size, we counted the number of xylem cells and phloem cells in each vascular bundle and measured the size of their area (Figure 1h–k, Table S1). To our surprise, the cell number increased in each vascular bundle of the *hdt1* mutant compared to the wild-type plant in the T3 stage. The numbers of xylem cells and phloem cells in the *hdt1-1* mutant were 22.0% and 18.4% greater than those of the wild type, respectively (Figure 1n). Identical phenotypes were confirmed in another mutant allele (*hdt1-2*) (Figure 1c,f,j,n). A complementation experiment was performed to confirm that the *hdt1* mutant phenotype was attributable to the loss of *HDT1* function (Figure 1c,g,k–n). Morphological analysis showed that cell number and stem thickness were recovered in the complementation transgenic (CT) lines.

These results showed that *HDT1* affected cell proliferation during vascular development, and thus inhibited the number of xylem cells and phloem cells. However, we also found that the sizes of the xylem cells in the *hdt1* mutant were smaller than in the wild type, and therefore, we further tested the cell size using transmission electron microscopy (TEM).

### 2.3. Xylem Cellular Structure in the hdt1 Mutant

**Figure 2:**
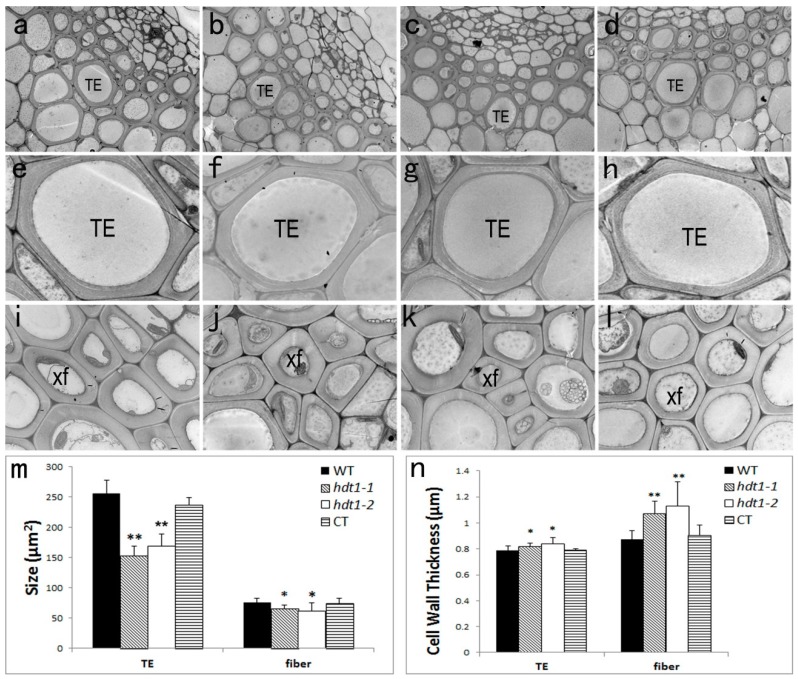
TEM of fiber cells and tracheary elements of the *hdt1* mutant, the CT line, and wild-type plants. Fiber cells and tracheary elements from the T3 stage were sectioned and examined under TEM. (**a**,**b**,**i**) Xylem cells in wild-type plants. (**a**) Overview of xylem cells. High-magnification images of tracheary elements (**e**) and fiber cells (**i**) in wild-type plants are shown. (**b**,**f**,**j**) Xylem cells in *hdt1-1* plants. (**b**) Overview of xylem cells. High-magnification images of tracheary elements (**f**) and fiber cells (**j**) in *hdt1-1* plants are shown. (**c**,**g**,**k**) Xylem cells in *hdt1-2* plants. (**c**) Overview of xylem cells. High-magnification images of tracheary elements (**g**) and fiber cells (**k**) in *hdt1-2* plants are shown. (**d**,**h**,**l**) Xylem cells in the CT lines. (**d**) Overview of xylem cells. High-magnification images of tracheary elements (**h**) and fiber cells (**l**) in the CT lines are shown. (**m**) Sizes of tracheary elements (TEs) and fiber cells were measured in wild-type, *hdt1-1*, *hdt1-2*, and CT lines. (**n**) The cell wall thickness of tracheary elements and fiber cells is shown in both wild-type and *hdt1* plants. The cell wall thickness was measured as the mean value of several symmetrical points across the cell wall of each cell. Scale bars = 20 μm (**a**–**d**) and 5 μm (**e**–**l**). The tracheary elements (TEs) and xylary fiber (XF) are indicated. (Data are means of three replicates ± SD. **, *p*-value < 0.01; *, *p*-value < 0.05).

Xylem cells were derived from cambium and displayed significant differences between *hdt1* mutants and wild-type plants. By examining the differences in detail at the cellular level using TEM, we confirmed that the size and cell wall thickness of the xylem cells was also altered in the *hdt1* mutants compared with the wild type in the T3 stage (Figure 2). We measured the area of xylem cells, including tracheary elements (TE) and fiber cells, and found that xylem cells were slightly smaller in *hdt1* mutant plants, particularly in TEs (Figure 2a–l). The cell size of TEs (153.2 ± 16.16 μm^2^) in the *hdt1-1* mutant was significantly decreased by 40.25% compared with the wild type (256.4 ± 21.38 μm^2^) (*p*-value < 0.01), and the size of fiber cells was slightly decreased by 13.79% in the *hdt1-1* mutant (65.0 ± 6.61 μm^2^) compared with the wild-type plants (75.4 ± 6.84 μm^2^) (Figure 2m). TEs and fiber cells are characterized by thicker secondary cell walls with three layers, which can be used to distinguish them from parenchyma cells [31]. Cell wall thickness also appeared to be altered in fiber cells and TEs (Figure 2e–g,i–k). Therefore, we measured the cell wall thicknesses of fiber cells (1.07 ± 0.10 μm) and TEs (0.82 ± 0.02 μm) in the *hdt1-1* mutant as well as that of fiber cells (0.87 ± 0.07 μm) and TEs (0.79 ± 0.04 μm) in the wild-type plants. Compared with wild-type plants, the cell wall thickness of fiber cells in the *hdt1-1* mutant was increased by 22.48%, while the cell wall thickness of TEs in the *hdt1-1* mutant was increased by 4.07% (Figure 2n). The mutant *hdt1-2* and the complementation transgenic line were tested in the same way. The cell size of TEs and fiber cells in the *hdt1-2* mutant was decreased by 34.01% and 19.10% compared with wild-type plants, respectively, While the cell wall thickness of TEs and fiber cells in the *hdt1-2* mutant was increased by 6.87% and 29.36% compared with wild-type plants, respectively. The size and cell wall thickness of TEs and fiber cells in CT lines did not alter compared with that of the wild-type plants (Figure 2a–d). This observation suggests that *HDT1* was involved in the development of xylem in vascular tissues and may affect secondary wall formation in xylem cells.

### 2.4. Lignin Analysis

**Figure 3:**
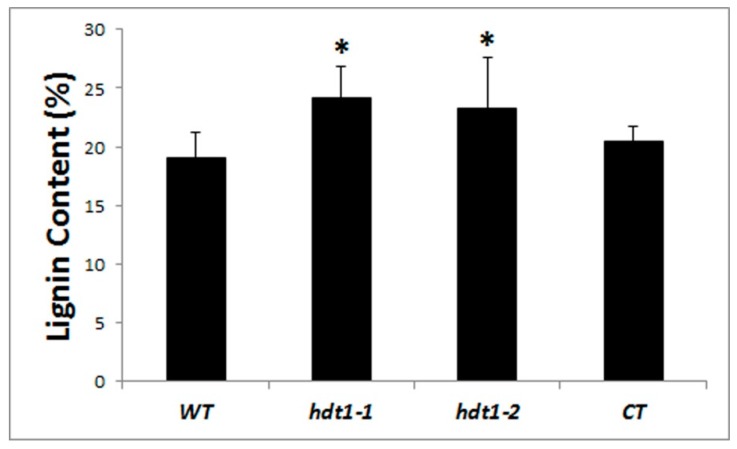
Lignin content in the stems of wild type, *hdt1-1*, *hdt1-2*, and the complementation transgenic line at the T3 stage. (*, 0.01< *p*-value < 0.05).

Lignin deposition happened during the formation of secondary cell walls in TEs and fiber cells. Because secondary cell wall thickness increased in both type of cells in the *hdt1* mutant, we analyzed the lignin content of the stem using the classical acetyl bromide method to determine whether the lignin content was altered in the *hdt1* mutant. Our results showed that the lignin content of the *hdt1* mutant was significantly increased compared with the wild type. The lignin contents were 24.14% and 23.24% in the *hdt1-1* and *hdt1-2* mutant stems, respectively, compared to 19.00% in the stem of the wild-type plant. The lignin content was 20.49% in the CT line (Figure 3), similar to that of wild-type plants. This result suggested that HDT1 might be involved in the lignin biosynthesis pathway, leading to an increased lignin content and secondary cell wall in the *hdt1* mutant.

### 2.5. Changes in Expression of Genes Affected by HDT1 During Stem Development

**Figure 4:**
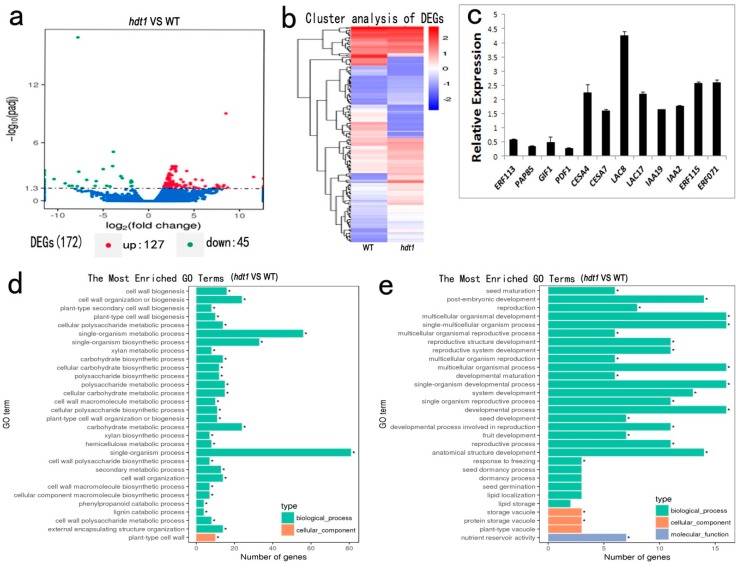
Transcriptomic analysis of differentially expressed genes. (**a**) Genes with significant differences in expression are indicated in the volcano plot by red dots (upregulation), green dots (downregulation), and blue dots (no significant difference). (**b**) Hierarchical cluster analysis of differentially expressed genes (*hdt1* versus the wild type). Red color indicates high expressed genes and blue color indicates low expressed genes in each sample. (**c**) Real-time quantitative analysis of differentially expressed genes (hdt1 mutant vs. the wild type). (**d**,**e**) GO enrichment analysis of the significantly upregulated (**d**) and downregulated (**e**) genes (*hdt1* mutant vs. the wild type; *, *p* < 0.005, log_2_ fold change >1 or <−1) in the T3 stage. GO terms were sorted based on the *p*-value.

To examine gene expression regulated by *HDT1* in vascular tissue development in *Arabidopsis*, we employed transcriptomic analysis using RNA-seq from the stems of wild-type and *hdt1* mutant plants at the T3 stage. In total, 172 genes were identified with significantly different expression levels (log_2_ fold change >1 or <−1, and corrected *p*-value < 0.005). Among them, 127 genes were upregulated and 45 genes were downregulated (Figure 4a). To visualize gene expression patterns in *hdt1* mutants, we performed hierarchical cluster analysis of differentially expressed genes (DEGs, Figure 4b) and tested the statistical enrichment of DEGs using the KOBAS software. We also performed gene ontology (GO) term enrichment analysis for each cluster to further characterize the functions of the DEGs in the *hdt1* mutant (Figure 4d,e).

GO term enrichment analysis was performed to determine which biological processes are involved in the stem development of *hdt1* mutants. Among the upregulated genes, those belonging to the “plant-type cell wall” cellular component constituted the only significantly represented term. Components of the plant cell wall were mainly organized into cellulose, hemicelluloses, and lignin. Upregulated DEGs in cellular processes included pathways related to cell wall organization or biogenesis, single-organism metabolic processes, carbohydrate biosynthetic processes, cellular polysaccharide metabolic processes, lignin catabolic processes, and xylan metabolic/biosynthesis processes, which were represented in *hdt1* mutants (Figure 4d). The formation of the secondary wall in fiber cells and TEs is the main process occurring in the vascular tissue during stem development, and therefore, we analyzed the genes involved in its biosynthesis (Table 1). Cellulose synthase (CESA) proteins are responsible for the synthesis of cellulose [32]. Two genes encoding the cellulose synthase complex (*IRX3/CESA7* and *IRX5/CESA4*) were upregulated in the *hdt1* mutant. Meanwhile, lignin is a phenolic component and can be synthesized by the phenylpropanoid biosynthesis pathway. Four genes related to the “phenylpropanoid metabolic process” were upregulated (Figure 4d), including *phenylalanine ammonia-lyase 1* (*PAL1*), *laccase 17* (*LAC17*), *LAC8*, and *LAC4/IRX12*. PAL catalyzes the first step from phenylalanine to cinnamic acid in the phenylpropanoid pathway, and downregulation of the *PAL* gene leads to a reduced lignin content [33]. Laccase is involved in the polymerization of lignin, and mutation of *LAC17* and *LAC4* leads to decreased lignin deposition in the stem of *Arabidopsis* [34,35]. In addition, seven genes involved in xylan biosynthesis were upregulated. Among them, *IRX9* encodes glycosyltransferases responsible for xylan backbone synthesis [36], while *xylan alpha-glucuronosyltransferase 1* (*GUX1*) encodes a glucuronosyltransferase responsible for adding GlcA substitutions to xylan [37], and *galacturonosyltransferase 12* (*GAUT12*) is required for xylan and lignin deposition [38] (Table 1). In addition, many genes associated with secondary cell wall components and genes involved in stem growth were upregulated. For instance, fasciclin-like arabinogalactan protein (FLA) belongs to the AGP protein family and may be involved in plant growth and development. *FLA11* and *FLA12* were upregulated in the *hdt1* mutant, and *AtFLA11*, *PtFLA11*, and *ZeFLA11* are all involved in the formation of secondary cell walls [39,40,41]. *AtFLA11* and *AtFLA12* mutations affect the strength and elasticity of plant stems [42].

Many transcription factors involved in lignification were upregulated in the *hdt1* mutant, including MYB, IAA, and ERF transcription factors. PtMYB4 and EgMYB2 have been reported to be involved in lignification by binding to AC elements in the promoter region of lignin biosynthesis genes [43], thus regulating secondary wall formation and lignin deposition [12]. Different MYB transcription factors can act as activators or repressors in lignification [44]. It has been reported that MYB87 affects cell wall organization and remodeling [45]. Other transcription factors related to hormone signaling were upregulated in the *hdt1* mutant. Auxin is a key signaling molecule involved in secondary xylem formation and cambial cell division [46,47]. Among auxin-related genes, two *AUXIN/INDOLE-3-ACETIC ACID* (*AUX/IAA*) factors, *IAA2* and *IAA19*, were upregulated in *hdt1* mutants. Two *ETHYLENE RESPONSE FACTOR* (*ERF071* and *ERF115*) genes encoding AP2/ERF transcription factors are involved in the ethylene signaling pathway and regulation of cell division in the cambium during vascular development. It has been reported that *erf018/erf109* double mutant displays reduced cell numbers in the vascular bundle [48]. The transcription factor, ERF115, acts as the rate-limiting factor for quiescent center cell division, controlling stem cell longevity [49].

In total, 45 genes were significantly downregulated in the *hdt1* mutant (Figure 4a). Based on GO term enrichment analysis, pathways related to “nutrient reservoir activity” and “seed maturation and post-embryonic development” were represented (Figure 4e). One downregulated gene, *PROTODERMAL FACTOR 1* (*PDF1*), is expressed in the protoderm of organ primordia [50], and is responsible for fiber cell differentiation in cotton [51], while the functions of other genes were largely unreported.

We verified the DEGs obtained from the transcriptomic analysis through qRT-PCR analysis. Twelve genes were selected based on high fold change and/or functional relevance (Figure 4c). Close agreement of the fold changes between qRT-PCR and the transcriptomic data indicated that the transcriptomic data were reliable (Pearson’s correlation coefficient of 0.9214) (Table S3).

Overall, transcriptomic analysis demonstrated that the upregulated genes in various pathways of secondary wall synthesis were related to thicker secondary cell walls in *hdt1* mutant plants. In addition, many DEGs were related to cell division, which may lead to cell number alteration in *hdt1* mutants. Altered expression of these genes may result in altered cambium cell proliferation and increased cell wall thickness in the *hdt1* mutant.

## 3. Discussion

Previous studies suggested that histone acetylation affects gene expression in the regulatory network and induces pleiotropic effects in the development of *Arabidopsis* [25]. This study investigated the expression pattern and mechanism of HDACs underlying stem vascular tissue formation through anatomical and transcriptomic analyses. Our results revealed that *HDT1* is involved in regulating cell division and secondary growth-related genes, and thus influences the cell number, cell size, and deposition of cell wall components during vascular tissue development.

### 3.1. HDT1 Participates in Regulation of Secondary Cell Wall Deposition during Xylem Development

Our data revealed that loss of HDT1 increases lignin content while also causing cell wall thickening during xylem development, which suggests that HDT1 regulates secondary cell wall formation and lignin biosynthesis. It has been reported that acetylation changes can affect gene expression and that the loss of HDAC (HDA19) causes developmental defects in *Arabidopsis*, including dwarfism, premature senescence, abnormal leaves, delayed flowering, and other phenotypes [28]. In this study, qRT-PCR and transcriptomic data revealed that many genes related to the biosynthesis of secondary wall components, including cellulose, lignin, and xylan, were upregulated in the *hdt1* mutant. Some of these upregulated genes affected the quantities of certain cell wall components. For example, CESA proteins are responsible for the synthesis of cellulose, and hence increases in *CESA4* and *CESA7* expression affect the content of cellulose [52]. Overexpression of several MYB genes from *Antirrhinum*, *Arabidopsis*, and grape (*Vitis vinifera*) has been shown to cause an alteration in lignin biosynthesis [53,54]. MYB87 may function as a regulator of genes affecting cell wall organization and remodeling [45]. PAL directly participates in the lignin biosynthesis pathway [12], and downregulation of PAL leads to reduced lignin content [33]. These genes are found among the upregulated DEGs in the *hdt1* mutant, which is consistent with the increased lignin content. Since lignin is mainly deposited in fibers and tracheary elements, TEM also showed that the cell wall in these cells became thicker. Therefore, HDT1 may affect cell wall deposition by regulating these related genes.

### 3.2. HDT1 Regulates Cell Division in Vascular Cambium

Cambium has meristem characteristics and thus can continue to proliferate or differentiate, a capability that plays an important role in the processes of root and stem growth. Our results showed that the number of xylem and phloem cells increased in the *hdt1* mutant. Through changes in the level of acetylation or altered expression of related genes, the number and morphology of cells can be changed. In principle, the increase in meristem cell number due to hyperacetylation could be caused either by an increased rate of cell division in meristematic cells or by delayed differentiation. Meanwhile, treatment with trichostatin A (TSA), an inhibitor of certain zinc-binding motif-containing histone deacetylases [55]. A previous report showed that increased levels of histone acetylation following TSA treatment delay cell differentiation in the root meristem, resulting in a greater number of meristem cells [56]. In our study, the absence of *hdt1* led to an increase in xylem cell numbers. Meanwhile, our qRT-PCR and transcriptomic data showed that many hormone-related genes, including *GA2ox8*, *IAA19*, and *IAA2*, were upregulated in *hdt1* mutants. Gibberellin (GA) can control root cell proliferation [57]. In *hdt1/2i*, upregulation of *GA2ox2* may lead to a decrease in the GA level, affecting the number of root meristem cells [27]. In addition to GA, the plant growth regulator auxin also controls cell identity, cell division, and cell expansion. In roots, an auxin gradient is clearly associated with patterns of cell proliferation and elongation observed along the apical–basal axis [58]. Overexpression of *AtIAA19* in *Arabidopsis* promotes the growth of roots, leaves, and stems [47]. Therefore, we propose that *HDT1* might also be involved in regulation of cambium activity, leading to increased xylem cell numbers.

However, we still do not know how *HDT1* regulates transcription of these genes. Histone deacetylation mediated by HDACs is recognized as playing a role in the regulation of gene expression and biological processes and is important in transcription inhibition and/or gene silencing. HD2 proteins mediate transcriptional repression by catalytic activity and decrease the acetylation level globally or specifically in plants [17]. Compared with the wild type, plants overexpressing *HDA19* showed phenotypic differences due to decreases in acetylated histone 3 (H3) [59]. The overexpression of *OsHDAC1* reduced the level of acetylated H4 significantly, which was accompanied by accelerated growth and alterations in plant architecture [60]. Histone deacetylases directly regulate gene transcription at a specific locus. It has been reported that *AtHD1* (*AtHDA19*) regulates gene expression during leaf and flower development, and gene activation is associated with increased acetylation levels at specific loci in the vicinity of promoters [61]. The HDAC proteins (HDT1/HDT2) have been found to repress the expression of *GA2OX2* by negatively regulating the acetylation level of *GA2OX2* in roots [27]. Based on our study, we speculate that the acetylation levels of *hdt1* mutants may affect morphological changes by regulating the expression of specific genes during vascular development. Acetylation thus influences stem growth by regulating cell proliferation and expanding secondary metabolite deposition in *Arabidopsis*.

Meanwhile, HDACs can also recruit other transcription repressor proteins as a complex to the promoter of target genes, thus repressing gene transcription. For example, HDA19 interacts with HSL1 to repress the expression of seed maturation genes. In the *hda19* mutant, the increased expression of seed maturation genes was correlated with histone hyperacetylation of chromatin [62]. HDT701 could bind to the promoter region of defense-related transcription factors, such as *MAPK6* and *WRKY53*, and negatively regulate their expression [63]. Whether increased expression of secondary growth genes in stems are caused by site-specific histone hyperacetylation or by recruiting other repressors needs to be determined by chromatin immunoprecipitation (ChIP) assays and bimolecular fluorescence complementation (BiFC), respectively.

In summary, *HDT1* may directly or indirectly affect gene expression during vascular tissue formation and is involved in vascular development and secondary metabolite deposition. Histone deacetylase might mediate chromatin structure changes and play an important role in regulating the division and differentiation of the vascular tissues.

## 4. Materials and Methods

### 4.1. Plant Materials and Growth Conditions

*Arabidopsis thaliana* (L.) Heynh Columbia was used as the wild type. The T-DNA insertion mutant seeds, AT3G44750 (GABI_355H03 and GABI_768H10), were obtained from the Arabidopsis Biological Resource Center. Transgenic *Arabidopsis* seeds created through complementation were obtained from a laboratory at Wageningen University [27]. The seedlings were sown in peat moss-enriched soil, vernalized for 5 days at 4 °C in the dark, and grown at 22 ± 3 °C for 1 to 2 months under a 14-h light/10-h dark cycle. PCR amplification was performed to identify homozygous plants using the following primers: *HDT1*-1-LB, 5′-ATATTGACCATCATACTCATTGC-3′. *HDT1*-1-LP, 5′-CGGCTTCGTATTAAAACCCTC-3′. HDT1-1-RP, 5′-GCCTTTGGTTTAGCTACAGCC-3′. And *HDT1*-2-LB, 5′-ATATTGACCATCATACTCATTGC-3′. *HDT1*-2-LP, 5′-CATCAATCGTGAAGCCTTG-3′. HDT1-2-RP, 5′-GTTGCAATATTCTCACA-3′.

### 4.2. Stem Growth Phenotype Analyses

The growth state of *Arabidopsis* was recorded in a phased way to examine the height and growth of the stem. We collected material based on the developmental stage and modified as previously described [64]. The growth state of the stem was divided into three stages. At the T1 stage, with bolting, the primary stem has begun to form. At the T2 stage, the plant has bolted and is flowering. At the T3 stage, all siliques have formed with only very few apical buds still present and the stems have turned yellow. More than 20 plants at various stages were measured in each line, including wild type, *hdt1* mutant, and the complementation transgenic line. Each sample was analyzed three times. All measurements and statistics were done using Origin8.0 and ImageJ2x Software.

### 4.3. RT-PCR and Quantitative Real-Time PCR (qRT-PCR)

HDT1 expression in various *Arabidopsis* tissues was assessed by semi-quantitative RT-PCR. Stems, roots, and leaves of *Arabidopsis* at the T3 stage were collected. According to the manufacturer’s instructions (Aidlab, Beijing, China), total RNA was isolated and first-strand cDNA synthesis was performed using a FastQuant Reverse Transcription Kit (Tiangen Biotech, Beijing, China). Actin was used as a reference gene with the actin-F/R primers: 5′-CGTATGAGCAAGGAGATCAC-3′ and 5′-CACATCTGTTGGAAGGTGCT-3′. HDT1 forward primers 5′-AGAAGAGCCTACACCTAAGAAGC-3′ and reverse primer 5′-TGAGACTTGACTGGCCGACT-3′ were used to amplify *HDT1*. The conditions for PCR were as follows: 95 °C for 5 min; 30 cycles at 94 °C for 10 s, 59 °C for 20 s, and 72 °C for 20 s; and 72 °C for 10 min. The intensity of PCR products detected by agarose gel electrophoresis indicated the gene expression level.

qRT-PCR analyses of the differentially expressed genes in stems at the T3 stage were performed using 2× SYBR Green qPCR mix (Thermo Scientific, Carlsbad, CA, USA on an iQ5 Multicolor Real-Time PCR detection system (Bio-Rad, Hercules, CA, USA). Primer information is shown in Table S2. The RNA of *hdt1* and wild-type *Arabidopsis* thaliana stems was extracted, and cDNA was synthesized as described above. The PCR conditions were as follows: 94 °C for 15 min; 40 cycles at 94 °C for 30 s, 55 °C for 20 s, and 72 °C for 20 s. Then, the solubility curves of the reaction were calculated, and the reaction was carried out at 95 °C for 30 s, at 65 °C for 30 s, and slowly raised to 95 °C with an increment of 0.5 °C/s. Error bars represent the standard error of independent triplicate assays. Data were analyzed using iQ5 software, and differences in gene expression were calculated using the 2^−ΔΔ*C*t^ method [65]. Each sample was analyzed three times.

### 4.4. RNA-Sequencing and Analysis of Differentially Expressed Genes

Stems without flowers and leaves from wild-type and *hdt1* mutant plants at the T3 stage were collected in triplicate. Total RNA was isolated using TRIzol reagent (Invitrogen, San Diego, CA, USA according to the manufacturer’s protocol. The Illumina Genome Analyzer (HiSeq 2000, Illumina, San Diego, CA, USA was used to sequence the samples. The gene expression level was calculated using reads per kb per million reads (RPKM) [66]. To analyze the differences between the wild-type and *hdt1* mutant plants, the DEGseq R package (1.12.0) was used. *p*-values were adjusted using the Benjamini method. A corrected *p*-value < 0.005 and |log2Ratio| >1 were set as the threshold for significantly different expression. Gene ontology (GO) enrichment analysis of differentially expressed genes was carried out using the GOseq R package, in which gene length bias was corrected [67]. The GO term (corrected *p*-Value < 0.05) was considered to be significantly enriched in differentially expressed genes. The data were deposited in the National Center for Biotechnology Information Gene Expression Omnibus database (http://www.ncbi.nlm.nih.gov/geo/query/acc.cgi?acc=GSE121407) under accession number GSE121407.

### 4.5. Microscopy

For histological analysis, we collected the first basal node of stems immediately above the uppermost rosette leaf and fixed at the T1, T2, and T3 stages (Figure S1) from wild-type, complementation transgenic line, and *hdt1* T-DNA insertion mutant plants in FAA (75% ethanol:acetic acid:formaldehyde, 90:5:5) for 36 h at 4 °C and then stored them in 75% ethanol. The stems were dehydrated with an alcohol gradient (75%, 85%, 95%, and 100% ethanol, 50 min each step), the stem was prestained with 0.1% Safranin O in ethanol, the ethanol was replaced with dimethylbenzene, and the samples were then embedded in paraffin (Sigma). Samples were cut in 8-μm thick transverse continuous slices with a Leica RM2016 microtome and dewaxed in dimethylbenzene, then dyed again in 0.1% Safranin O water solution for 12 h. These samples were rinsed three times with water and sealed with resin when they were dried. We observed these samples under a Leica DCF500 microscope. Micrographs of sections from five specimens at the T3 stage were analyzed from each line. Six vascular bundles in each specimen were counted. The number of xylem and phloem cells was counted, and the cross-sectional area was measured using ImageJ2x and each sample repeated three times. Statistical differences were determined using Student’s *t*-test.

### 4.6. Transmission Electron Microscopy (TEM)

The first basal segments of stems were collected for TEM at the T3 stage. The samples were stored in 0.25% glutaraldehyde, and then rinsed three times with PBS buffer. The samples were incubated in 1% osmic acid for 3 h, and then rinsed three times with PBS buffer. The samples were dehydrated with an alcohol gradient (75%, 85%, 95%, and 100% ethanol, 30 min each step), the ethanol was then replaced with propylene oxide, and the samples were embedded in low viscosity (Spurr’s) resin. For observation of subcellular structures, ultrathin sections (70 nm) were obtained with a UC6 ultramicrotome (Leica). Image acquisition was performed using an H-7650 transmission electron microscope (Hitachi) at 80 kV and an 832 charge-coupled device camera (Gatan, Warrendale, PA, USA. The materials were the same as those used for paraffin sections. Samples were collected from five plants in each line at the T3 stage, and each line was repeated three times. Approximately 60 cells, including fiber cells and tracheary elements, were counted in each sample. ImageJ2x software was used to calculate the cell size and cell wall thickness. Statistical differences were determined using Student’s *t*-test.

### 4.7. Lignin Analysis

The stems of the *hdt1* mutant, complementation transgenic line, and wild-type plants were harvested at the T3 stage, frozen immediately in liquid nitrogen, and freeze dried. The stem samples were milled to a fine powder, and then dried in a 100 °C oven. These materials were extracted with 75% ethanol at 28 °C for 12 h, and dissolved in ethanol:methylbenzene (2:1). The extracted samples were vacuum dried, and all the samples were sifted through a 100-mesh screen to preserve the samples. The samples (4–6 mg) were put into a reagent bottle, and 7.5 mL of glacial acetic acid, 2.5 mL of acetyl bromide, and 400 μL of perchloric acid were added successively to the bottle. Samples were incubated in a 70 °C water bath for 1 to 2 h and 2 M sodium hydroxide was added to stop the reaction, then glacial acetic acid was added to make a 50 mL final volume. Under the same conditions, the solution without experimental material was used as a control. The OD_280_ was measured using an ultraviolet spectrophotometer. The Klason technique was used to measure the lignin content [68]. Samples were collected from five wild-type plants and five *hdt1* mutant plants at the T3 stage. Each sample was analyzed three times.

## Figures and Tables

**Table 1:** Expression patterns of selected vascular tissue related genes.

Functional Category	AGI No.	Putative ID	^a^ Fold Change	*p*-Value
Upregulated Genes				
Transcription factors	AT2G47520	*ERF071*	8.1782	1.64 × 10^−4^
	AT5G07310	*ERF115*	3.0198	1.43 × 10^−4^
	AT3G15540	*IAA19*	1.7525	3.15 × 10^−4^
	AT3G23030	*IAA2*	1.6792	2.14 × 10^−4^
	AT4G37780	*MYB87*	8.6901	2.31 × 10^−4^
	AT2G40750	*WRKY54*	1.9848	9.54 × 10^−5^
	AT5G22570	*WRKY38*	5.8852	1.43 × 10^−5^
	AT1G25560	*TEM1*	1.8477	3.59 × 10^−5^
	AT2G30400	*OFP2*	3.0713	5.39 × 10^−5^
	AT2G30395	*OFP17*	6.2226	1.37 × 10^−4^
Lignin synthesis	AT5G01040	*LAC8, Laccase-8*	7.5751	2.12 × 10^−4^
	AT5G60020	*LAC17*	2.4538	1.69 × 10^−4^
	AT2G38080	*LAC4/IRX12*	2.5538	4.39 × 10^−8^
	AT5G39580	*PER62, Peroxidase 62*	7.1886	2.65 × 10^−4^
	AT2G37040	*PAL1, Phenylalanine ammonia-lyase 1*	1.7664	9.34 × 10^−5^
Xylan synthesis	AT2G37090	*IRX9, Beta-1,4-xylosyltransferase 9*	2.5472	2.33 × 10^−6^
	AT3G18660	*GUX1, Xylan alpha-glucuronosyltransferase 1*	2.5645	1.87 × 10^−7^
	AT5G54690	*GAUT12, Galacturonosyltransferase 12*	2.1985	5.86 × 10^−6^
	AT2G38320	*TBL34, Protein trichome birefringence-like 34*	3.9005	1.96 × 10^−4^
	AT3G55990	*Protein ESKIMO 1*	2.3088	5.61 × 10^−6^
	AT3G15050	*IQ-domain 10*	1.9627	9.72 × 10^−5^
Cellulose synthesis	AT5G44030	*CESA4, Cellulose synthase A catalytic subunit 4*	2.0603	8.00 × 10^−6^
	AT5G17420	*CESA7*	1.7013	1.34 × 10^−4^
Others related to cell wall formation	AT3G13520	*AGP12, Arabinogalactan peptide 12*	2.4194	2.27 × 10^−5^
	AT5G10430	*AGP4*	2.3787	1.72 × 10^−6^
	AT5G03170	*FLA11*	2.044	1.12 × 10^−5^
	AT5G60490	*FLA12, Fasciclin-like arabinogalactan protein 12*	1.9173	4.39 × 10^−5^
	AT1G41830	*SKS6*	2.9462	1.12 × 10^−7^
	AT4G08685	*SAH7, Pollen Ole e 1 allergen and extensin family protein*	1.9925	3.94 × 10^−5^
	AT5G15630	*COBL4, COBRA-like protein 4*	1.9612	3.83 × 10^−5^
	AT5G57220	*CYP81F2, Cytochrome P450, family 81, subfamily F, polypeptide 2*	1.7666	1.33 × 10^−4^
	AT1G62440	*LRX2, Leucine-rich repeat/extensin 2*	3.7927	2.62 × 10^−5^
	AT1G09610	*GXM1, Glucuronoxylan 4-O-methyltransferase 1*	2.9384	3.01 × 10^−7^
	AT2G32990	*AtGH9B8, Endoglucanase 11*	2.6682	3.45 × 10^−5^
	AT1G19940	*AtGH9B5*	1.9237	3.02 × 10^−4^
	AT5G16190	*CSLA11, Mannan synthase 11*	1.8223	2.52 × 10^−4^
	AT1G02640	*BXL2, Beta-D-xylosidase 2*	2.0764	4.82 × 10^−6^
	AT3G62020	*GLP10, Germin-like protein subfamily 2 member 4*	2.1733	9.13 × 10^−6^
	AT5G66920	*SKU5 similar 17*	2.4182	1.60 × 10^−4^
	AT1G45130	*Beta-galactosidase 5*	1.9347	2.25 × 10^−4^
Cell differentiation	AT1G04040	*HAD superfamily, subfamily IIIB acid phosphatase*	1.7168	2.42 × 10^−4^
Other	AT2G01280	*MEE65, Cyclin/Brf1-like TBP-binding protein*	4.5845	3.76 × 10^−5^
	AT4G21200	*GA2ox8, Gibberellin 2-beta-dioxygenase 8*	2.539	4.19 × 10^−5^
	AT1G20850	*XCP2, Xylem cysteine proteinase 2*	3.1489	1.45 × 10^−4^
*Downregulated Genes*				
Transcription factors	AT5G13330	*ERF113*	−1.9415	1.73 × 10^−5^
	AT1G02065	*SPL8, Squamosa promoter-binding-like protein 8*	−4.2595	1.71 × 10^−4^
Cell wall formation	AT3G22640	*PAP85, Cupin family protein*	−10.417	1.01 × 10^−5^
Cell cycle	AT1G53490	*HEI10, E3 ubiquitin-protein ligase CCNB1IP1 homolog*	−1.9356	1.50 × 10^−4^
Cell proliferation	AT5G28640	*GIF1, GRF1-interacting factor 1*	−4.6295	1.19 × 10^−4^
Other	AT1G24793	*LPXC4, N-acetylglucosamine deacetylase 5*	−2.919	7.19 × 10^−6^
	AT2G42840	*PDF1, Protodermal factor 1*	−5.894	1.37 × 10^−4^

^a^ Fold change: fold change between *hdt1* and wild type. Values mean log_2_ fold ratio. The data source comes from TAIR (http://www.arabidopsis.org/), NCBI (https://www.ncbi.nlm.nih.gov/) and UniProt (http://www.uniprot.org/).

## Supplementary Materials

Supplementary materials can be found at https://www.mdpi.com/1422-0067/20/14/3452/s1.

## Abbreviations

HDAC	Histone deacetylase
HD2	HD-tuins
HAT	Histone transferase
RPD3	Reduced potassium dependency 3
sir2	Silent information regulator 2
RT-PCR	Reverse transcription-polymerase chain reaction
qRT-PCR	quantitative real-time PCR
GA	Gibberellin
TEM	Transmission electron microscopy
GO	Gene Ontology
RPKM	Per kb per million reads
TE	Tracheary elements
DEGs	Differentially expressed genes
CESA	Cellulose synthase
CT	Complementation transgenic
WT	Wild type
CESA	Cellulose synthase
PAL1	Phenylalanine ammonia-lyase 1
LAC	Laccase
FLA	Fasciclin-like arabinogalactan protein
AUX/IAA	AUXIN/INDOLE-3-ACETIC ACID
GUX1	Xylan alpha-glucuronosyltransferase 1
ERF	ETHYLENE RESPONSE FACTOR
PDF1	PROTODERMAL FACTOR 1
TSA	Trichostatin A
H3	Histone 3
ChIP	Chromatin immunoprecipitation
BiFC	Bimolecular fluorescence complementation
