# Supplementary material for: Histone Deacetylase HDT1 is Involved in Stem Vascular Development in Arabidopsis
Source: Int J Mol Sci. 2019 Jul 13;20(14):3452. doi: 10.3390/ijms20143452 (PMC6678272; doi:10.3390/ijms20143452)
Supplement: Supplementary file 1 [file ijms-20-03452-s001.pdf]

Supplementary materials

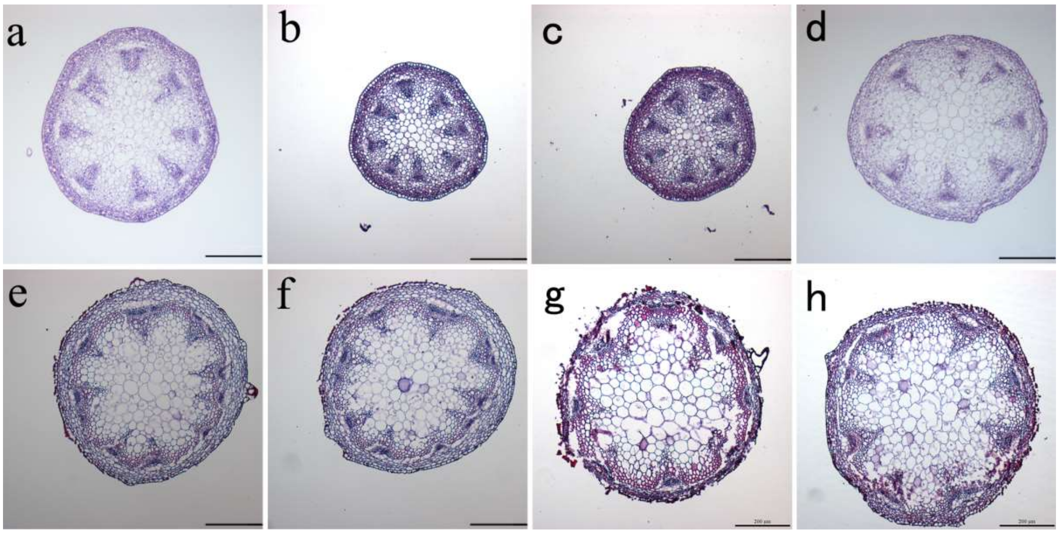

Figure S1. (**a–d**) Plants growth status at T1 stage. (**a**) is wild-type (WT), (**b**) is GABI_355H03 (*hdt1-1*), (**c**) is GABI_768H10 (*hdt1-2*) and (**d**) is CT. (**e–h**) Plants growth status at T2 stage. (**e**) is wild-type (WT), (**f**) is GABI_355H03 (*hdt1-1*), (**g**) is GABI_768H10 (*hdt1-2*) and (**h**) is CT. Scale bars = 200 μm (**a**–**h**)

Table S1. Area analysis of stem in WT and *hdt1*

**Area**	**WT**	***hdt1***	**Difference (*hdt1* vs. WT)**	***p*-Value**
Pith (mm^2)	0.3194 ± 0.0197	0.1923 ± 0.0115	−39.82%	$p < 0.01$
Xylem (mm^2)	0.1222 ± 0.0118	0.0899 ± 0.0031	−26.43%	$0.01 < p < 0.01$
Phloem (mm^2)	0.0432 ± 0.0028	0.0394 ± 0.0023	−9.05%	$0.01 < p < 0.05$

The analyses were performed based on WT and *hdt1* stems at T3 stage. Data shown are average ± SD. *p*-values were determined by Student's *t* test ($n \geq 10$).

Table S2. Primer information used for reverse transcription-polymerase chain reaction (RT-PCR)

**Primer Name**	**Sequence(5′-3′)**
AtActin2F	5' CATCCTCCGTCTTGACCTTGC 3'
AtActin2R	5' CAAACGAGGGCTGGAACAAG 3'
AT5G13330-F	5' TAAATCGGATCAACATCAACCAG 3'
AT5G13330-R	5' CGAAAGTCCCGAGCCAGA 3'
AT3G22640-F	5' GGTAGGGTTTGGTATTTATGCG 3'
AT3G22640-R	5' TTTGGCTCGTGAACAGTTTCT 3'
AT5G28640-F	5' TTCAACGCAACCTAATGTACCTAG 3'
AT5G28640-R	5' AATCATCCCACCACCAGCA 3'
AT2G42840-F	5' TTCTGTTGCTTCCGTAAGGTTT 3'
AT2G42840-R	5' GGAGTTGAAGGAGAAGGGTCGT 3'
AT5G17420-F	5' TTCCAGCCATCTGTCTCCT 3'
AT5G17420-R	5' CTAACTCCGCTCCATCTCAA 3'
AT5G44030-F	5' GCCGTTTGTCTTCTCACCG 3'
AT5G44030-R	5' TGATGCTTACACCACTCCACC 3'
AT5G01040-F	5' CCAGGCGGATGGGTTGT 3'
AT5G01040-R	5' CGGAGTTGGCCCGTTTT 3'
AT5G60020-F	5' CGATTCCTTGCAGATAACCCAG 3'
AT5G60020-R	5' CCAAGCCATCCTCAGACCC 3'
AT3G23030-F	5' GAAGAATCTACACCTCCTACCAA 3'
AT3G23030-R	5' CTCCGTCCATACTCACTTTCA 3'
AT3G15540-F	5' ATGATGATCTAGCCTTTGCTCTTG 3'
AT3G15540-R	5' TTCGCAGTTGTCACCATCTTTC 3'
AT5G07310-F	5' AATCAACAAGATCCTAACCCACC 3'
AT5G07310-R	5' GCTGCCCACTTTCCCCAT 3'
AT2G47520-F	5' AAGAAGCGTAAACCCGTCTCA 3'
AT2G47520-R	5' TGGCCTCTGCCTTATCCCT 3'

Table S3. qRT-PCR corroboration of differentially expressed genes.

Gene ID		Change Fold	
		Transcriptomic Data	qRT-PCR
AT5G13330	*ERF113*	−1.9415	-1.3577
AT3G22640	*PAP85*	−10.417	-2.3427
AT5G28640	*GIF1*	−4.6295	-1.8356
AT2G42840	*PDF1*	−5.894	-2.6308
AT5G44030	*CESA4*	2.0603	1.9431
AT5G17420	*CESA7*	1.7013	1.1501
AT5G01040	*LAC8*	7.5751	2.7969
AT5G60020	*LAC17*	2.4538	1.9139
AT3G15540	*IAA19*	1.7525	1.2624
AT3G23030	*IAA2*	1.6792	1.4578
AT5G07310	*ERF115*	3.0198	2.1857
AT2G47520	*ERF071*	8.1782	2.1967
Pearson correlation coefficient		0.921433	

Fold-change: Log_2 (*hdt1*/wild type).
